# Cardiovascular risk assessment in persons with HIV in the developing world: comparing three risk equations in a cohort of HIV-infected Thais

**DOI:** 10.1186/1758-2652-13-S4-O40

**Published:** 2010-11-08

**Authors:** N Edwards-Jackson, SJ Kerr, HV Tieu, J Ananworanich, SM Hammer, K Ruxrungtham, P Phanuphak, A Avihingsanon

**Affiliations:** 1 Columbia University College of Physicians & Surgeons, New York, USA; 2 The HIV Netherlands Australia Thailand Research Collaboration, The Thai Red Cross AIDS Research Center, Bangkok, Thailand; 3 South East Asia Research Collaboration with Hawaii, Bangkok, Thailand, Bangkok, Thailand

## Purpose

There are growing concerns of cardiovascular disease in HIV-infected individuals and in developing countries, such as Thailand. We described the ten-year risk of coronary heart disease (CHD) in a Thai HIV-infected cohort using 3 cardiovascular risk equations, and assessed the level of agreement between their predictions.

## Methods

Cross-sectional analysis of data from 785 Thai subjects followed prospectively in the HIV Netherlands Australia Thailand Collaboration (HIV-NAT) cohort study from 1996-2009. Cardiovascular risk factor history, along with relevant laboratory and clinical data, was collected at follow-up clinic visits. Ten-year risks of CHD were calculated using the Framingham, Ramathibodi-Electricity Generating Authority of Thailand (Rama-EGAT), and Data-collection on Adverse Effects of Anti-HIV Drugs (D:A:D) risk equations.

## Results

**Figure 1:**
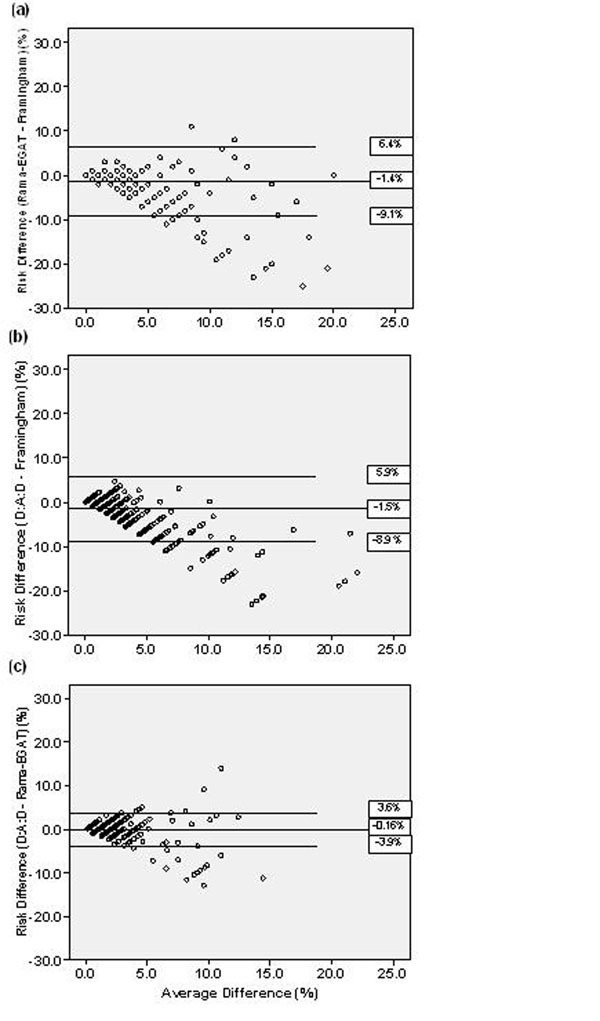


Mean age was 41.0 years; 55% of subjects were male. Mean CD4 count was 569 cells/mm3 after a mean of 7.7 years on anti-retroviral therapy. The prevalence of cardiovascular risk factors was low, with the most common risk factor being low high density lipoprotein (36.3%). The prevalence of high cardiovascular risk scores (defined as ten-year risk of CHD ≥10%) was also low: 9.9%, 2.1%, and 0.8%, by the Framingham, Rama-EGAT, and D:A:D scoring systems, respectively. Only 8 subjects (1.0%) had a history of CHD. Bland-Altman plots revealed that the Framingham risk score was, on average, 1.4% (S.D. 3.9%) higher than the Rama-EGAT and 1.5% (S.D. 3.7%) higher than the D:A:D (Figure 1a,b). The limits of the difference showed that the Framingham could be as high as 9.1% above or as low as 6.4% below the Rama-EGAT, and as high as 8.9% above or as low as 5.9% below the D:A:D. The Bland-Altman plot comparing the D:A:D and Rama-EGAT equations (Figure 1c) demonstrated a smaller average difference (-0.16%) and narrower limits of the difference (-3.9% and 3.5%). All differences were most pronounced for subjects with higher average risk scores.

## Conclusions

The predicted cardiovascular risk in this HIV-infected Thai cohort was relatively low. The Framingham equation predicted the highest cardiovascular risks, which is consistent with its known tendency to over-predict risk in Thais. The agreement between the Rama-EGAT and D:A:D risk scores suggests that both equations may be appropriate estimators of cardiovascular risk in this and other developing world populations with low background risk.
